# Awareness and factors associated with reported intake of folic acid-fortified flour among women of reproductive age in Ifakara, Morogoro region, Tanzania: a cross-sectional study

**DOI:** 10.1186/s40795-019-0324-5

**Published:** 2019-11-25

**Authors:** Ipyana Frank Mwandelile, Rose Mpembeni, Ahmed Abade, Susan F. Rumisha, Julius J. Massaga, Rogath Kishimba

**Affiliations:** 1 Muhimbili University of Health and Allied Sciences (MUHAS), P.O.Box 65015, Dar es- salaam, Tanzania; 2 Field Epidemiology and Laboratory Training Programme (FELTP), P.O.Box 71286, Dar es- salaam, Tanzania; 3 National Institute for Medical Research, P.O.Box 9653, 3 Barack Obama Drive, 11101 Dar es Salaam, Tanzania

**Keywords:** Intake, Folic acid, Fortification, Flour, Women of reproductive age, Ifakara, Tanzania

## Abstract

**Background:**

Folic acid fortification of staple foods has been in place in many countries for over two decades. Studies have shown that folic acid fortification can significantly reduce incidence of neural tube defects. Tanzania adopted a mandatory fortification policy for commercially-produced wheat and maize flour in 2011. We determined factors influencing intake of folic acid-fortified flour among women of reproductive age (WRA).

**Methods:**

We conducted a cross-sectional study among WRA during March–April 2017 in Ifakara Town Council, Morogoro region. Multistage cluster sampling was used to select study participants. We used a questionnaire to capture information on demographics, awareness of folic acid, awareness of existence of folic acid fortified flour in community and intake of folic acid fortified flour. Intake was defined as reported consumption of folic acid fortified flour products at least once within 7 days before interview. Univariate, bivariate, and multivariable logistic analyses were done to evaluate factors associated with intake of folic acid fortified flour.

**Results:**

The median age of the 698 participating WRA was 30 years (range: 18–49). Awareness of folic acid and folic acid fortified flour was 6.9% (95% CI: 5.2–9.0%) and 7.5% (95% CI: 5.7–9.6%), respectively. Consumption of folic acid fortified flour was 63.3% (95% CI: 59.7–66.8%). Independent factors associated with intake included being employed (aOR = 1.91; 95% CI: 1.19–3.06), having no children (nulliparity) (aOR = 2.59; 95% CI: 1.36–4.95) or having 1–4 children (aOR = 1.98; 95% CI: 1.17–3.33) (vs. 5 or more children), and folic acid awareness (aOR = 2.53; 95% CI: 1.30–4.92).

**Conclusion:**

Folic acid fortified flour was used by most respondents in our study despite low awareness of existence of folic acid fortified flour in the community. Being employed, having fewer than five children, and folic acid awareness were independent factors associated with intake. We recommend scaling up of mandatory flour fortification program and doing further studies on blood folate level among women of reproductive age in Ifakara to assess fortification program effectiveness.

## Background

Globally 86 countries have regulation to mandate fortification of at least one industrially milled cereal grain [1]. Food fortification, such as fortification with vitamins A, B, and D, iron, and iodine has a long history in developed countries for control of various micronutrient and vitamin deficiencies in the population [2]. Folic acid fortification of staple foods has been in place for over two decades after several studies demonstrated that it can help prevent neural tube defects (NTDs) [3]. Pregnancies are often unplanned, even in high-income countries, rendering it difficult to target interventions during the peri-conception period [3]. Fortification of staple foods with folic acid allows all women to access this nutrient throughout their reproductive years and thus reduce the risk of NTDs without active behavioral change [3]. To date, there are more than fifty countries with mandatory regulations for fortification of wheat flour with folic acid [4].

Tanzania passed a policy for mandatory fortification of commercial wheat and maize flour in 2011 [5]. Wheat flour is fortified with folic acid, iron, vitamin B12, zinc, thiamin, riboflavin, niacin and vitamin A, while maize flour is fortified with folic acid, iron and vitamin B12. Most common wheat flour available in the market is produced by a few large companies with fortification capability. In contrast, maize flour is produced by many small-scale millers who have limited resources and few incentives to fortify their products [5]. Therefore, practically all wheat flour available on the market is fortified whereas only some maize flour is fortified.

Reports indicate that the Ifakara Town Council has one of the highest rates of birth defects, particularly NTDs, in Tanzania [6]. It is expected that the requirements for wheat/maize flour fortification with folic acid will lead to adequate consumption of folic acid, which in turn could reduce the NTD burden; these studies remain to be done. However, there is little information available on the intake of folic acid fortified flour among women of reproductive age (WRA) in Tanzania since introduction of the fortification policy. We assessed awareness of folic acid and folic acid fortified flour existence, reported intake and factors influencing intake among WRA in Ifakara.

## Methods

### Study design, setting and subjects

We conducted a community-based cross-sectional study during March and April, 2017 in Ifakara Town Council, Kilombero district, Morogoro region, Tanzania. Ifakara Town Council is one of nine councils of Morogoro region with nine wards, 16 villages, and 64 hamlets. It has an estimated population of 106,424 people and 25,542 WRA (aged 18–49 years), with an average household size of 4.5 persons [7, 8]. All WRA were eligible for participating in the study with the exclusion of those who were unable to communicate/talk or those who had resided in the council for < 6 months.

### Sampling

Sample size was calculated using Kish Leslie formula (1965) for cross sectional studies. A design effect of 1.8, margin of error of 5%, and non-response rate of 10% were used to calculate the sample size of 690.

We selected study participants using multistage cluster sampling. First, five wards were selected by simple random sampling from nine wards, followed by selection of nine villages as first-stage units. The total population of villages was used to calculate the required number of clusters per village. Thirty clusters (hamlets/streets) were selected as second-stage units by probability proportional-to-size. Twenty-four households from each cluster were selected for the study as third-stage units. Households included in the study were selected randomly using the WHO EPI cluster sampling method [9] whereby a central location was identified and a direction selected at random by spinning a pen. All houses were counted in a straight line following this direction until the edge of the hamlet/street was reached. One of the counted houses was picked at random to create the starting point of the study. The second house visited was one that was nearest to the front door of the first household visited, followed by the next household until the sample size was reached.

Only one eligible woman from each household was enrolled in the study. If more than one woman was eligible, one was selected randomly. If no member of the household was eligible, the next household was visited.

### Data collection

Data were collected using administered questionnaires for the household by trained interviewers (Additional file 1).

Information gathered included respondent age, education, income, marital status, occupation, parity, awareness of folic acid, awareness of existence of folic acid fortified flour in community and intake of folic acid-fortified flour. Intake was defined as reported consumption of folic acid fortified flour products at least once within 7 days prior the survey.

For folic acid fortified maize flour, we considered intake only among respondents who could remember the maize brand consumed. For folic acid fortified wheat flour, we considered intake among respondents who took wheat flour products from any brand because all wheat brands are fortified.

### Data analysis

Data were entered and analyzed using Epi Info 7 and STATA 13. We conducted a univariate analysis to describe general characteristics of respondents and calculate response frequencies; continuous variables were summarized by measures of central tendency and dispersion (median and range).

Ascertainment of independent factors influencing intake of folic acid-fortified flour among WRA was done by multiple logistic regression analysis through stepwise forward elimination; while controlling for potential confounders and adjusting for hamlet/streets clustering effect. All variables with a significance level of *p* ≤ 0.2 in bivariate analysis, including conventional confounders such as age, were added in the multiple logistic regression model.

## Results

### Sociodemographic and economic characteristics and folic acid awareness

We visited 710 households with eligible WRA, 698 of them participated in the study; response rate 98.3%. Reasons for non response included refusal and absentism. Their median age was 30 years; range 18–49 years. Most had primary education (564, 80.8%), were unemployed (501, 71.7%), and were married or cohabiting (420, 60.2%). More than half (365, 52.3%) lived in households with five or more persons.

Of the 698 participants, 48 (6.9%; CI: 5.2–9.0%) reported having heard of folic acid. Among these 48 women, only five (10.4%) knew that folic acid could prevent some birth defects.

Awareness of folic acid was reported more frequently among women with secondary education or more (13.4%, *n* = 18) than those with primary education or less (5.3%, *n* = 30) (*p* < 0.01) (Table 1).

**Table 1:** Awareness of folic acid by demographic and socioeconomic characteristics in Ifakara, 2017 (*N* = 698)

Awareness of folic acid				*P* value
Characteristic	No	Yes	cOR (95%CI)	
	n (%)	n (%)		
Age group				
18–24	178 (91.3)	17 (8.7)	Ref	0.49*
25–35	254 (93.7)	17 (6.3)	0.70 (0.35–1.41)	
36–49	218 (93.9)	14 (6.0)	0.67 (0.32–1.40)	
Education				
Primary or less	534 (94.7)	30 (5.3)	Ref	0.003*^a^
Secondary or more	116 (86.6)	18 (13.4)	2.49 (1.33–4.69)	
Marital status				
Married/ Cohabiting	388 (92.4)	32 (7.6)	Ref	0.41^
Divorced/separated/Widowed	85 (96.6)	3 (3.4)	0.43 (0.13–1.44)	
Single	177 (93.2)	13 (6.8)	0.89 (0.46–1.74)	
Occupation				
1	185 (93.9)	12 (6.1)	0.84 (0.43–1.65)	0.61*
2	465 (92.8)	36 (7.2)	Ref	
Income/month				
< 150,000 Tshs	114 (94.2)	7 (5.8)	Ref	0.67*
150,000+ Tshs	444 (93.1)	33 (6.9)	1.21 (0.52–2.81)	
Household size				
< 5	312 (93.7)	21 (6.3)	Ref	0.57*
≥ 5	338 (92.6)	27 (6.4)	1.19 (0.66–2.14)	
Parity				
0	100 (92.6)	8 (7.4)	1.70 (0.49–5.87)	0.72^
1–4	465 (92.8)	36 (7.2)	1.65 (0.57–4.75)	
5+	85 (95.5)	4 (4.5)	Ref	

Missing values for income

*cOR* Crude odds ratio, *CI* Confidence interval

Occupation: 1 = Employed (including Self-employed/business), 2 = Unemployed (including Peasant/Maid/housewife/student, Tshs = Tanzanian shillings

* Pearson chi square test ^Fisher exact test ^a^Statistically significant

### Awareness of community existence and intake of folic acid fortified flour

Only 52 (7.5%; CI: 5.7–9.4%) of 698 participants reported having heard of folic acid-fortified flour. There was no statistically significant difference in having heard of folic acid fortified flour by socioeconomic or demographic characteristics (Table 2). Almost two-thirds (442; 63.3%) of women reported having consumed folic acid-fortified flour brands at least once within the 7 days before the survey. Of these, three (0.7%) had consumed folic acid fortified maize flour brands only, and 436 (98.6%) had used folic acid fortified wheat flour brands only. The remaining three (0.7%) reported that they had consumed both folic acid fortified maize and wheat flour brands.

**Table 2:** Awareness of existence of folic acid fortified flour in community by -demographic and economic characteristics in Ifakara, 2017 (*N* = 698)

Awareness of folic acid fortified flour existence				
Characteristic	No	Yes	cOR (95% CI)	P value
	n (%)	n (%)		
Age group				
18–24	178 (91.3)	17 (8.7)	Ref	0.56*
25–35	250 (92.3)	21 (7.8)	0.88 (0.45–1.72)	
36–49	218 (93.9)	14 (6.0)	0.67 (0.32–1.40)	
Education				
Primary or less	526 (93.3)	38 (6.7)	Ref	0.14*
Secondary or more	120 (89.5)	14 (10.5)	1.61 (0.84–3.08)	
Marital status				
Married/ Cohabiting	391 (93.1)	29 (6.9)	Ref	0.79*
Divorced/separated/ Widowed	81 (92.1)	7 (7.9)	1.66 (0.49–2.75)	
Single	174 (91.6)	16 (8.4)	1.24 (0.66–2.34)	
Occupation				
1	181 (91.9)	16 (8.1)	1.14 (0.62–2.11)	0.67*
2	465 (92.8)	36 (7.2)	Ref	
Income per month				
< 150,000 Tshs	108 (89.3)	13 (10.7)	Ref	0.16*
150,000+ Tshs	444 (93.1)	33 (6.9)	0.62 (0.31–1.22)	
Household size				
< 5	305 (91.6)	28 (8.4)	Ref	0.36*
≥ 5	341 (93.4)	24 (6.6)	0.77 (0.43–1.35)	
Parity				
0	102 (94.4)	6 (5.6)	0.81 (0.25–2.63)	0.66*
1–4	461 (92.0)	40 (7.9)	1.20 (0.49–2.92)	
5+	83 (93.3)	6 (6.7)	Ref	

Missing values for income

*cOR* Crude odds ratio, *CI* Confidence interval

Occupation: 1 = Employed (including Self-employed/business), 2 = Unemployed (including Peasant/Maid/housewife/student, Tshs = Tanzanian shillings

* Pearson chi square test

### Factors influencing intake of folic acid fortified flour

The odds of folic acid fortified flour intake were two times higher for employed women compared to unemployed women [aOR = 1.91, 95% CI: (1.19–3.06)]. (Table 3).

**Table 3:** Factors associated with reported intake of folic acid fortified flour in Ifakara, 2017

Intake of fortified flour			
Characteristics	n (%)	cOR (95% CI)	aOR(95% CI)^a^
Age group			
18–24	129 (66.2)	Ref	
25–35	169 (62.4)	0.85 (0.58–1.25)	1.05 (0.72–1.52)
36–49	144 (62.1)	0.84 (0.56–1.25)	1.23 (0.72–2.11)
Education			
Primary or less	342 (60.6)	Ref	
Secondary or more	100 (74.6)	1.82 (1.19–2.78)	1.46 (0.89–2.37)
Marital status			
Married/ Cohabiting	261 (62.1)	Ref	
Divorced/separated/ Widowed	51 (57.9)	0.84 (0.53–1.34)	0.86 (0.43–1.72)
Single	130 (68.4)	1.32 (0.92–1.90)	1.07 (0.71–1.60)
Occupation			
Employed	146 (74.1)	1.98 (1.37–2.87)	1.91 (1.19–3.06)^**b**^
Unemployed	296 (59**.**1)	Ref	
Household size			
< 5	203 (60.9)	Ref	
≥ 5	239 (65.5)	1.21 (0.89–1.65)	1.32 (0.98–1.78)
Parity			
0	77 (71.3)	2.66 (1.45–4.88)	2.59 (1.36–4.95)^**b**^
1–4	322 (64.3)	1.92 (1.22–3.04)	1.98 (1.17–3.33)^**a**^
5+	43 (48.3)	Ref	
Aware (heard) of folic acid			
Yes	39 (81.3)	2.66 (1.26–5.60)	2.53 (1.30–4.92)^**b**^
No	403 (62)	Ref	
Aware (heard) of fortified flour			
Yes	37 (71.2)	1.47 (0.79–2.73)	–
No	405 (62.7)		

a: *p* = 0.01, b: *p* < 0.01

*aOR* adjusted odds ratio; *CI* confidence interval, *cOR* crude odds ratio;

^a^adjusted for age, education, marital status, occupation, household size, parity, heard of folic acid and heard of fortified flour

Moreover, nulliparous women were nearly three times more likely to have consumed folic acid fortified flour than those who had five children or more, [aOR =2.59; 95% CI: (1.36–4.95)]. Women who had 1–4 children had nearly twice the odds of having taken up folic acid fortified flour compared to those who had five or more children, [aOR = 1.98; 95% CI: (1.17–3.33)].

Women who were aware of folic acid had two and a half times greater odds of having consumed folic acid fortified flour than those who were not aware [aOR = 2.53, 95%CI: (1.30–4.92)] (Table 3).

## Discussion

This study was conducted to assess awareness of folic acid, awareness of existence of folic acid fortified flour in community, consumption of folic acid-fortified flour, and factors influencing intake among WRA in Ifakara, Morogoro region, Tanzania. Two third of participants reported consumption of folic acid fortified flour despite low awareness of its existence. Being employed, having fewer than five children, and folic acid awareness were independent factors associated with intake. These findings provide a snapshot of the level of folic acid fortified flour intake among WRA after initiation of a fortification program in Ifakara.

Folic acid awareness was low, far less than awareness reported in other studies done in Nepal, Honduras, Europe, USA, and New Zealand where awareness ranged from 40 to 98% [10–14]. Furthermore, our study found that only five (10.1%) respondents who were aware of folic acid knew that folic acid can prevent some birth defects. This is low compared to studies from Europe, New Zealand, and Honduras [12, 14, 15]. The low awareness could be due to the fact that in Tanzania, during antenatal visits folic acid is provided in combination with iron in a formulation known as “FEFO”, typically with health education on the prevention of anaemia alone.

Awareness of folic acid fortified flour was low as well, but higher than a report from a Northern Ugandan study where no woman was aware of existence of fortified foods [16]. The low level of awareness among WRA in Ifakara could be attributed to the fact that the fortification policy is relatively new in Tanzania, having been in practice for only 4 years. The fortification program was officially launched in 2013 involving various local and international actors and stakeholders [17, 18].

In our study, self-reported intake of folic acid fortified flour among WRA was 63.3%. Intake of commercially-produced folic acid fortified wheat flour was higher than folic acid fortified maize flour. This could be explained by the fact that all wheat flour brands available in the council were fortified, widely used for breakfast snacks, and produced by big industries, as opposed to maize flour which is milled by local small-scale millers within the district. Although all small-scale millers are encouraged to fortify maize flour, only a few do. In some parts of Tanzania, including Ifakara, an organization named “SANKU” provides fortification dosifiers, premix, and technical assistance to selected small- or medium-scale mills [19]. Currently there are three small-scale mills in Ifakara under this support. Therefore, we found only a few brands of folic acid fortified maize flour, one of which was predominant in the market. Other maize flour brands were available but not fortified.

This study revealed that employed women were almost two times as likely to have consumed folic acid fortified flour compared to unemployed women. The lower intake by unemployed persons in our study could either be due to low purchasing power that would not allow buying commercial foods, or due to the fact that they use self-produced foods in their home rather than commercial foods. In addition, employed women are more likely to be educated compared to unemployed women [20–22] and may be better positioned financially (stable income).

Another independent factor that influenced intake of folic acid fortified flour among WRA in Ifakara was parity (being nulliparous or having fewer than 5 children). This observation was contrary to our expectation, as we thought exposure would be high in multiparous women and would correspond with high intake of folic acid fortified flour. However, nulliparous women might be younger, more educated, and more financially sound than women with many children, and thus likely to consume fortified foods. Also women with fewer children are more likely to have small family size hence more chance of fortified flour intake through ability to manage and feed the small family.

Strength of this study is that it was conducted at the community level, and the relatively large sample size could be representative of the community. The other strength is high response rate.

However, the study had some limitations, including possible recall bias. We attempted to reduce this by collecting information on intake of folic acid-fortified flour within 7 days before the interview. Furthermore the study was cross-sectional whose results can not establish causality. Also respondents in the same cluster may assume similar characteristics which could have overestimated or underestimated intake or associations. This was encountered by adjusting for clustering effect during analysis. The study did not measure quantity of folic acid consumed in fortified flour products.

## Conclusion

Overall intake was reported by most of study participants despite low awareness of existence of fortified flour. This underscores the importance of mandatory fortification, which ensures that most WRA are reached, influencing reduction of the micronutrient deficiency and NTD burden. However, efforts to increase intake of folic acid-fortified flour among women and the general community are needed in Ifakara. We recommend ensuring availability of folic acid fortified flour by scaling up fortification programs to all maize flour small-scale mills. Moreover, we recommend doing blood folate level studies in Ifakara among women of reproductive age, since use of folic acid fortified flour has shown to raise blood folate level as evidenced by a study done by Noor et al. in urban Dar es Salaam, Tanzania [23]. This will help assessment of fortification program effectiveness.

## Supplementary information

**Additional file 1.** An English version questionnaire used to gather data for this study.

## Data Availability

The datasets used and/or analysed during the current study are available from the corresponding author on reasonable request.

## Abbreviations

CDC: Centers for Disease Control and Prevention
EPI: Expanded Program of Immunization
FA: Folic acid
FEFO: Iron and Folic
MOHCDGEC: Ministry of Health Community Development Gender Elderly and Children
MUHAS: Muhimbili University of Health and Allied Sciences
NBS: National Beural of Statistics
NTD: Neural Tube Defect
PPS: Probability Proportion to Size
TFELTP: Tanzania Field Epidemiology and Laboratory Training Program
TFNC: Tanzania Food and Nutrition Centre
USA: United States of America
WHO: World Health Organization
WRA: Women of Reproductive Age
