# Supplementary material for: Awareness and factors associated with reported intake of folic acid-fortified flour among women of reproductive age in Ifakara, Morogoro region, Tanzania: a cross-sectional study
Source: BMC Nutr. 2019 Nov 25;5:55. doi: 10.1186/s40795-019-0324-5 (PMC7050702; doi:10.1186/s40795-019-0324-5)
Supplement: Supplementary file 1 — Additional file 1. An English version questionnaire used to gather data for this study. [file 40795_2019_324_MOESM1_ESM.docx]

## Appendix 1c: QUESTIONNAIRE - English version

**AWARENESS AND REPORTED INTAKE OF FOLIC ACID FORTIFIED FLOUR AMONG WOMEN OF REPRODUCTIVE AGE IN IFAKARA-MOROGORO, 2017.**

**Questionnaire number_______**

**Date of Interview______________ Interviewer ID______**

**Ward____________________Village/hamlet___________________Cluster #____________**

**Now we are going to talk about folic acid and staple flours**

**SECTION A: KNOWLEDGE ON FOLIC ACID/FORTIFIED FLOUR**

1. Have you ever heard of folic acid? ^[1]^

1. Yes
2. No....................................If **No**/Don’t know/Not sure

skip to question number 4

1. Don’t know/Not sure

2. If **Yes**, what have you heard, read, or seen about folic acid? (*Circle all that apply*)

1. It can prevent some birth defects
2. It is a vitamin needed for cell growth
3. It is a type of acid found in some household products that should be avoided in pregnancy
4. It is good for pregnant women
5. It needs to be taken before pregnancy
6. It needs to be taken during pregnancy
7. It is good for health
8. Don’t remember
9. Others (specify)…………………………….

3. If **Yes** who gave you the information on folic acid? (*Circle all that apply*)

1. Health care providers
2. Magazine/news papers/books
3. Radio/television/internet
4. Family/friend

4. Have you ever heard of fortified flour?

1. Yes
2. No....................................If **No** skip to question number 6

5. If **Yes**, who gave you the information on fortified flours? (*Circle all that apply*)

1. Health care providers
2. Magazine/news papers/books
3. Radio/television/Internet
4. Family/friend

**SECTION B: REPORTED INTAKE OF FORTIFIED FLOUR**

**Now we are going to talk about maize flour**

**MAIZE FLOUR**

6. Does your household prepare foods using fortified maize flour?

1. Always
2. Regularly
3. Sometimes
4. Rarely
5. No, never....................................If **No** skip to question number 15
6. Don’t know/Not sure…………Skip to question number 11

7. Where does your household get fortified maize flour? *Circle all that apply*

1. Purchase
2. Get it from relatives/ friends
3. Receive from food aid
4. Others (specify)…………………………….

8. If your household purchase maize flour what is the brand of maize flour you have ever purchased? *Circle all that apply*

i. Kindole super sembe ii. Mzalendo super sembe iii. Mgaya super sembe iv. Super seki v. JC super sembe vi. M.K. super sembe vii. Master super sembe viii. Mwangaza super sembe ix. MC Super sembe x. P.M super sembe xi. Super sembe xii. Don’t remember xiii. Others (specify)…………………………….

9. In the past seven days have you eaten any of the fortified maize flour products (*like porridge and ugali/posho*?

1. Yes
2. No....................................If **No** skip to question number 16

10. If Yes, How many days have you eaten any of the fortified maize flour products in the last seven days? *____________________*

11. Do you currently have any maize flour in your household?

1. Yes
2. No....................................If **No/**Don’t know

skip to question number 17

1. Don’t know

12. If **Yes**, Could you show me the original package? *Observe on fortification logo*

1. Logo present
2. Logo absent

13. If **Yes** and packed in original package, *Observe on brand name*

i. Kindole super sembe ii. Mzalendo super sembe iii. Mgaya super sembe iv. Super seki v. JC super sembe vi. M.K. super sembe vii. Master super sembe viii. Mwangaza super sembe ix. MC Super sembe x. P.M super sembe xi. Super sembe xii. Others (specify)…………………………….(***Go to question 17***)

14. If not packed in original package, *Ask what was/is the brand of the flour?*

i. Kindole super sembe ii. Mzalendo super sembe iii. Mgaya super sembe iv. Super seki v. JC super sembe vi. M.K. super sembe vii. Master super sembe viii. Mwangaza super sembe ix. MC Super sembe x. P.M super sembe xi. Super sembe xii. Don’t remember xiii. Others (specify)…………………………….(***Go to question 17***)

15. What are the reasons for not preparing food using fortified maize flour in your household? *Circle all that apply*

1. Not available
2. Expensive
3. We don’t prefer fortified maize flour
4. We buy readymade products
5. We prepare flour at home/local mills
6. Buy unknowingly/don’t know flour is fortified
7. Others (specify)…………………………….

16. What are the reasons for not eating fortified maize flour products in the last seven days?

1. Not available
2. Expensive
3. I/we don’t prefer fortified maize flour
4. I/we bought readymade products
5. Prepared flour at home/local mills
6. Out of stock
7. Others (specify)…………………………….

**WHEAT FLOUR**

17. Does your household prepare foods using wheat flour?

1. Always
2. Regularly
3. Sometimes
4. Rarely
5. No, never....................................If **No** skip to question number 21
6. Don’t know/remember……………**Go to question 22**

18. Where does your household get wheat flour? *Circle all that apply*

1. Purchase
2. Get it from relatives/ friends
3. Receive from food aid
4. Others (specify)…………………………….

19. If your household purchase wheat flour what is the brand of wheat flour you have ever purchased? *Circle all that apply*

i. Azania ii. Pembe iii. Maisha iv. Safi v. Poa vi. Taifa vii. Azam viii. Nyati

ix. Jumbo x. Sunkist xi. Don’t know/remember xii. Others (specify)…………………………….**Go to question 22**

20. What are the reasons for not preparing food using wheat flour? *Circle all that apply*

1. Not available
2. Expensive
3. We don’t prefer fortified wheat flour
4. We buy readymade products
5. Buy unknowingly/don’t know flour is fortified
6. Others (specify)…………………………….

21. In the past seven days have you eaten any of the wheat flour products? (*like bread, buns, chapatti, cake etc*)

1. Yes
2. No.................................... If **No** skip to question number 24

22. If Yes, How many days have you eaten any of the wheat flour products in the last seven days? *___________________***Go to question 24.**

23. What are the reasons for not eating wheat flour products in the last seven days?

1. Not available
2. Expensive
3. I/we don’t prefer fortified wheat flour
4. I/we bought readymade products
5. Out of stock
6. Others (specify)…………………………….

24. Do you currently have any wheat flour in your household?

1. Yes
2. No....................................If **No/**Don’t know skip to question number 28
3. Don’t know

25. If **Yes**, Could you show me the original package? *Observe on fortification logo*

1. Logo present
2. Logo absent

26. If **Yes** and packed in original package, *Observe on brand name*

i. Azania ii. Pembe iii. Maisha iv. Safi v. Poa vi. Taifa vii. Azam viii. Nyati

ix. Jumbo x. Sunkist xi. Others (specify)…………………………….

27. If not packed in original package, *Ask what was/is the brand of the flour?*

i. Azania ii. Pembe iii. Maisha iv. Safi v. Poa vi. Taifa vii. Azam viii. Nyati ix. Jumbo x. Sunkist xi. Don’t remember xii. Others (specify)………

**Go to question 28**

**SECTION C: SOCIO-ECONOMC AND DEMOGRAPHIC CHARACTERISTICS**

28. How old are you now?....................... (Years)

29. What is the maximum level of education you attained? *Circle one response*

1. Informal/illiterate
2. Primary school
3. Secondary and high school
4. Diploma
5. University

30. What do you do to earn a living? *Circle one response*

1. Student
2. Peasant
3. employee
4. Self employed/business
5. Unemployed/Housewife/maid

31. What is your household estimated maximum monthly income?..............................................Tshs

32. What is your current marital status? *Circle one response*

1. Never married
2. Married
3. Cohabiting
4. Divorced/separated
5. Widowed

33. How many are you in your household, including you?.......................

34. What is your relationship with the head of household?

1. I am the head of household
2. I am the wife/ partner of head of household
3. I am a relative/child of head of household
4. I am a house girl/non-relative

35. Have you ever given birth to a child/children?

1. Yes
2. No....................................If **No** (***END HERE****)*

36. How many times have you given birth to children including (if any) those who died while seven months pregnant or more?...................

***Now we are going to specifically talk about your LAST pregnancy.***

37. During your last pregnancy, did you attend at antenatal clinic?

1. Yes
2. No....................................If **No** skip to question number 40

38. If Yes, in what month of pregnancy did you start attending antenatal clinic?__________

39. How many times did you attend at antenatal clinic? *__________________*

40. Did you plan your latest pregnancy with a health care provider?

1. Yes
2. No

**END**

Thank you for your participation

**References/Links to some questions in this questionnaire**

**1. Question 1:** [**https://www.ncbi.nlm.nih.gov/pmc/articles/PMC3643291/**](https://www.ncbi.nlm.nih.gov/pmc/articles/PMC3643291/)
